# The differences between pure and mixed invasive micropapillary breast cancer: the epithelial–mesenchymal transition molecules and prognosis

**DOI:** 10.1007/s10549-024-07384-w

**Published:** 2024-07-02

**Authors:** Ozden Oz, Resmiye Irmak Yuzuguldu, Ayse Yazici, Demet Kocatepe Cavdar, Cengiz Yilmaz, Mucteba Ozturk, Hilal Duzel, Duygu Gurel

**Affiliations:** 1 Department of Pathology, Izmir Bozyaka Training and Research Hospital, University of Health Sciences, Izmir, Turkey; 2 Department of Pathology, Mugla Training and Research Hospital, Mugla, Turkey; 3 Department of Pathology, Faculty of Medicine, Training and Research Hospital, Izmir Katip Celebi University, Izmir, Turkey; 4 Department of Medical Oncology, Izmir Bozyaka Training and Research Hospital, University of Health Sciences, Izmir, Turkey; 5 Department of General Surgery, Izmir Bozyaka Training and Research Hospital, University of Health Sciences, Izmir, Turkey; 6 Department of Public Health, Medical Faculty, Dokuz Eylul University, Izmir, Turkey; 7 Department of Pathology, Medical Faculty, Dokuz Eylul University, Izmir, Turkey

**Keywords:** Pure breast micropapillary carcinoma, Epithelial–mesenchymal transition (EMT), Prognostic parameters, Prognosis

## Abstract

**Purpose:**

Invasive micropapillary carcinoma (IMPC) of the breast is known for its high metastatic potential, but the definition of pure and mixed IMPC remains unclear.

This retrospective cohort study aims to investigate the prognostic significance of the micropapillary component ratio and the expression of critical molecules of epithelial–mesenchymal transition (EMT), including E-cadherin (E-cad), N-cadherin (N-cad), CD44s, and β-catenin (β-cat), in distinguishing between pure and mixed IMPCs.

**Methods:**

We analyzed 100 cases of locally advanced IMPC between 2000 and 2018 and excluded patients who received neoadjuvant chemotherapy. Pure IMPC was defined as having a micropapillary component of over 90%. A comprehensive recording of prognostic parameters was conducted. The IMPC areas were analyzed using the immunohistochemical (IHC) staining method on the microarray set for pure and mixed IMPC patients. Pearson's chi-square, Fisher’s exact tests, Kaplan–Meier analysis, and Cox proportional hazards analysis were employed.

**Results:**

The comparative survival analysis of the entire group, based on overall survival (OS) and disease-free survival (DFS), revealed no significant difference between the pure and mixed groups (*P* = 0.480, HR = 1.474 [0.502–4.325] and *P* = 0.390, HR = 1.587 [0.550–4.640], respectively). However, in the pure IMPC group, certain factors were found to be associated with a higher risk of short survival. These factors included skin involvement (*P* = 0.050), pT3&4 category (*P* = 0.006), a ratio of intraductal component (> 5%) (*P* = 0.032), and high-level expression of N-cad (*P* = 0.020). Notably, none of the risk factors identified for short OS in pure IMPC cases were observed as significant risks in mixed cases and vice versa. Furthermore, N-cad was identified as a poor prognostic marker for OS in pure IMPCs (*P* = 0.002).

**Conclusion:**

The selection of a 90% ratio for classifying pure IMPCs revealed significant differences in certain molecular and prognostic parameters between pure and mixed groups. Notably, the involvement of N-cadherin in the epithelial–mesenchymal transition (EMT) process provided crucial insights for predicting OS and DFS while also distinguishing between the two groups. These findings strongly support the notion that the pure IMPC subgroup represents a distinct entity characterized by unique molecular characteristics and behavioral patterns.

**Supplementary Information:**

The online version contains supplementary material available at 10.1007/s10549-024-07384-w.

## Introduction

According to global cancer statistics from 2020, breast cancer is the most commonly diagnosed cancer and the fifth leading cause of cancer-related deaths worldwide [1]. The rate of metastasis in breast carcinomas is higher in comparison to other cancer types, except for Malignant Melanoma [2]. Invasive micropapillary carcinoma (IMPC) of the breast is a distinct form of breast cancer characterized by tumor cells arranged in morula-like clusters or pseudopapillary structures without a fibrovascular core. These structures are found within empty stromal spaces [3–5]. The IMPC was first described in 1980 and later included in the WHO classification as IMPC, naming it for the breast by Siriaunkgul and Tavassoli in 1993 [5, 6].

It is widely acknowledged in the literature that IMPC has a high metastasis rate [6–9]. Furthermore, the presence of any proportion of IMPC component is considered an important predictive factor for lymph node metastasis [6, 10, 11]. While several publications indicate that IMPC has a poor prognosis [3, 5, 9, 11–13], there are also studies reporting that it does not have a different prognosis compared to invasive breast carcinoma of no special type (IBC-NST) [6, 14, 15]. In fact, some studies even suggest that IMPC has a better prognosis [7].

The diagnosis of IMPC is conducted through the evaluation of morphological features as well as the demonstration of an “inside out” staining pattern using the epithelial membrane antigen (EMA) immunohistochemical (IHC) analysis method, specifically targeting MUC1 [3, 16]. This distinctive staining pattern is recognized as evidence of “reverse polarity.” IMPC can generally be categorized as either pure or mixed based on the proportion of the micropapillary component. However, despite varying reports in the literature regarding the prognosis differences between IMPC and IBC-NST [5–7, 9, 15, 17], there is limited available data to differentiate between pure and mixed IMPC patients [10–13, 18, 19]. Consequently, the independence of pure IMPC as a distinct entity remains unknown.

The epithelial–mesenchymal transition (EMT) hypothesis is widely accepted as the primary explanation for the molecular mechanisms underlying invasion and metastasis, which are crucial hallmarks of cancer [20]. However, in the case of IMPC, a type of cancer known for its high metastatic capacity, there is a notable absence of studies in the existing literature that specifically examine the alterations of molecules implicated in the EMT process as it relates to the differentiation between pure and mixed tumor types.

EMT, an active process, is characterized by decreased adhesion molecule synthesis and increased mesenchymal protein synthesis. However, the specific diagnostic molecular markers still require clarification [21–24]. One crucial molecular finding is the substitution of E-cadherin (E-cad) with N-cadherin (N-cad) [25, 26]. Additionally, the transition from isoform variants (CD44v) of CD44 surface antigen (CD44s), a co-receptor involved in cellular signaling pathway regulation during EMT, to the standard isoform (CD44s) is considered significant [27]. β-catenin (β-cat), when bound to E-cad, forms a complex supporting adherent connection. However, in cells with impaired interactions with the surrounding tissue, CD44 inhibits E-cad expression, disrupting this complex and allowing β-cat to translocate to the nucleus, triggering the EMT process [28].

Our study aims to classify pure and mixed IMPC cases in our breast carcinoma cohort group diagnosed with IMPC, using the 90% criterion recommended in the 5th WHO classification. Furthermore, we aim to compare this classification with prognostic parameters to investigate whether the pure and mixed IMPC groups represent distinct entities. Additionally, we seek to examine the expression changes of E-cad, N-cad, β-cat, and CD44s in the EMT process in IMPC cases and assess their contribution to this distinction in a molecular prognostic manner. To the best of our knowledge, these approaches have not been extensively discussed in the literature, making our research pioneering in these aspects.

## Material and methods

### Patient population

#### Clinicopathological data

Firstly, the Ethics Committee of the University of Health Sciences, Izmir Bozyaka Education and Research Hospital approved using primary tissue samples in this study (Reference Number: 2022/64, Date: April 6, 2022). Secondly, in this retrospective cohort study, 4526 cases diagnosed with breast cancer between 2000 and 2018 in the Dokuz Eylul University, Medical Faculty, Izmir Bozyaka Education and Research Hospital, and Izmir Katip Celebi University’s Pathology Departments’ archive records were analyzed. Thirdly, 212 cases diagnosed with IMBC were investigated among these patients. Due to several reasons, including those who received neoadjuvant treatment (48), consultation cases (13), male gender (4), micro-metastasis (10), and no lymph node metastasis (37), 112 patients were excluded. Consequently, 100 cases, either pure or mixed type, who did not receive neoadjuvant treatment and had locally advanced and higher stages were included in the study. All processes performed in this study followed the Declaration of Helsinki (2013). The demographical and clinic data resources of the cases were obtained from the digital archive systems of each hospital.

#### Histopathologic and prognostic parameters examination

Four experienced pathologists re-examined all tumor-containing H&E-stained slide sections of 100 cases. The diagnosis was confirmed through the morphologic criteria for the histological classification of breast tumors outlined by the World Health Organization (WHO) and inspection of the reversal of the polarity pattern through typical staining with EMA (IHC) (E29, mouse monoclonal antibody, 790-4463; Roche). The pure-type IMPC cases were defined as constituting at least 90% of the micropapillary component of the tumor.

Age, primary tumor size, secondary tumor size (if applicable) tumor sizes, the rate of the ductal carcinoma in situ component (DCIS) (it was calculated by examining the entire tumor area), number of lymph node metastasis (LNM), size of the largest LNM, overall survival (OS), and disease-free survival (DFS) were recorded numerically.

Perineural invasion (PNI), lympho-vascular invasion (LVI), pericapsular invasion of lymph node, recurrence (local or distant), skin involvement, resection margin tumor positivity, modified Scarff–Bloom–Richardson score, and its subcomponents, type of surgery performed, the clinical and pathological stage, and the intensity of tumor-infiltrating lymphocyte (TIL) were recorded categorized. The TIL scoring system is that Score 0: no infiltrating lymphocytes; Score 1: mild increase of infiltrating lymphocytes in the tumor nest or stroma; Score 2: increased infiltrating lymphocytes interwoven with tumor tissue; and Score 3: prominent infiltrating lymphocytes separate or incorporated in tumor tissue. Scores 0 and 1 were accepted as low, and scores 2 and 3 were high TIL [29]. The clinical and pathological stage was determined according to the World Health Organization’s Tumor Classification of Breast Tumors 5th edition.

#### Survival data

The OS time was calculated as the period between the date of the Tru-cut biopsy and the date of death or the last observation for living patients. Similarly, the DFS time was determined as the duration from the Tru-cut biopsy to the recurrence time for both living and deceased patients. The patients’ follow-up data were last updated in November 2022.

### Tissue microarrays

One representative area of micropapillary breast carcinoma from mixed IMPC and pure IMPC was carefully selected from formalin-fixed paraffin-embedded tumor tissues in our cohort. Subsequently, each cancer tissue area, with a diameter of 0.6 mm, was selected from a donor block and was transferred to an empty space of the recipient paraffin block. Non-micropapillary areas from mixed tumors were not evaluated in our study. The production of tissue microarrays (TMAs) was carried out following the previously described protocol by Kononen et al. [30]

### Immunohistochemical method

Estrogen receptor (ER), progesterone (PR), Ki-67, and human epidermal growth factor receptor 2 (HER2/CerbB2) biomarkers were used in the molecular classification. The investigation of E-cad, N-cad, CD44, beta-catenin biomarkers, and molecular classification markers was performed on the tissue microarray set using the IHC staining method. 4-micron-thick sections were obtained for each microarray block. All sections were deparaffinized according to standard procedures and, after rehydrated, underwent heat-induced antigen retrieval for 5 min in the Dako pressure cooker at 121 °C using pH 9 Dako Target Retrieval Solution™ (Agilent, CA, U.S.A., #S2367). The antibodies used, along with their sources, clones, and dilution rates, are listed in Table 1. The Dako autostainer with the Dako DAB detection kit was employed to reveal antibody binding. The list and using condition of the antibodies are displayed in Supplementary Table.

**Table 1:** Comparative pathological characteristics of pure and mixed invasive micropapillary carcinomas (IMPCs)

Parameters	Mixed	Pure	*P* value
*Mortality*			
Death	25 (33.3%)	4 (16.0%)	0.098^3^
Alive	50 (66.7%)	21 (84.0%)	
*Mean age (years)*			
< 60	42 (56.0%)	12 (48.0%)	0.487^3^
≥ 60	33 (44.0%)	13 (52.0%)	
*Tumor size*			
≤ 20 mm	31 (42.5%)	8 (32.0%)	0.356^3^
> 20 mm	42 (57.5%)	17 (68.0%)	
*Number of the metastatic lymph node*			
< 4	45 (60.8%)	16 (64.0%)	0.777^3^
≥ 4	29 (39.2%)	9 (36.0%)	
Largest lymph node size*	16.5 ± 9.0	17.4 ± 13.2	0.837^2^
Intraductal component ratio*	17.8 ± 21.3	2.0 ± 4.6	**< 0.001**^2^
Number of the tumoral mass			1.000^1^
One mass	66 (88.0%)	22 (88.0%)	1.000^1^
Multifocal mass	9 (12.0%)	3 (12.0%)	
*Pericapsular invasion*			
Absent	36 (48.6%)	14 (60.9%)	0.306^3^
Present	38 (51.4%)	9 (39.1%)	
*Recurrence*			
Absent	57 (76.0%)	13 (52.0%)	**0.023**^3^
Present	18 (24.0%)	12 (48.0%)	
*Distant metastasis*			
Absent	57 (76.0%)	17 (68.0%)	0.430^3^
Present	18 (24.0%)	8 (32.0%)	
*Skin involvement*			
Absent	66 (88.0%)	19 (76.0%)	0.194^1^
Present	9 (12.0%)	6 (24.0%)	
*Pathological category*			**0.013**^1^
pT1&2	74 (98.7%)	21 (84.0%)	0.013^1^
pT3&4	1 (1.3%)	4 (16.0%)	
*Clinical stage*			
IIA&IIB	45 (60.0%)	13 (52.0%)	0.483^3^
III&IV	30 (40.0%)	12 (48.0%)	
*Modified Scarff–Bloom–Richardson histological grading*			
1&2	38 (51.4%)	12 (48.0%)	0.772^3^
3	36 (48.6%)	13 (52.0%)	
*Tubule formation*			
1&2	24 (32.4%)	2 (8.0%)	**0.016**^3^
3	50 (67.6%)	23 (92.0%)	
*Nuclear grade*			
1&2	31 (41.9%)	9 (36.0%)	0.604^3^
3	43 (58.1%)	16 (64.0%)	
*Mitosis*			
1&2	34 (70.1%)	14 (66.0%)	0.207^3^
3	22 (29.7%)	11 (44.0%)	
*Surgical margin*			
Negative	70 (94.6%)	23 (92.0%)	0.641^1^
Positive	4 (5.4%)	2 (8.0%)	
*Tumor-infiltrating lymphocytes*			
Low	17 (27.9%)	0 (0.0%)	**0.002**^1^
High	44 (72.1%)	25 (100.0%)	
*Vascular embolus*			0.904^3^
Absent	49 (65.3%)	16 (64.0%)	
Present	26 (34.7%)	9 (36.0%)	
*Nerve invasion*			
Absent	61 (82.4%)	23 (92.0%)	0.343^1^
Present	13 (17.6%)	2 (8.0%)	
*Lympho-vascular embolus*			
Absent	28 (37.3%)	12 (48.0%)	0.346^3^
Present	47 (62.7%)	13 (52.0%)	
ER*	69.5 ± 36.4	57.4 ± 43.3	0.415^2^
PR*	47.5 ± 39.0	42.0 ± 41.9	0.595^2^
Ki67*	32.8 ± 23.6	36.7 ± 21.1	0.362^2^
*Cerb2 IHC*			
Score < 3	65 (86.7%)	18 (72.0%)	0.123^1^
Score = 3	10 (13.3%)	7 (28.0%)	
*BCAT*			
Low	14 (18.7%)	3 (12.0%)	0.550^1^
High	61 (81.3%)	22 (88.0%)	
*CD44*			
Low	40 (53.3%)	17 (68.0%)	0.200^3^
High	35 (46.7%)	8 (32.0%)	
*N CAD*			
Low	50 (66.7%)	20 (80.0%)	0.208^3^
High	25 (33.3%)	5 (20.0%)	
*ECAD*			
Low	25 (33.3%)	5 (20.0%)	0.208^3^
High	50 (66.7%)	20 (80.0%)	
Overall survival*	83.6 ± 61.3	63.0 ± 41.1	0.277^3^
Disease-free survival*	77.8 ± 61.8	56.9 ± 45.3	0.208^3^

^1^Fisher’s exact test was used

^2^Mann–Whitney *U* test was used

^3^Pearson Chi-square test was used

*Mean value

#### Interpretation of immunohistochemical staining

The positive control tissues included the following: normal small intestine tissue for CD44, normal tonsil tissue for E-cad, normal appendix for β-cat, normal liver for N-cad, positive breast cancer tissue for CerbB2, and normal breast tissue for ER, PR, Ki-67, and P53.

CD44, E-cad, N-cad, and β-cat IHC staining were considered acceptable if membranous staining was observed. Nuclear staining was considered positive for ER, PR, and the proportion of positive cells was recorded as a percentage of the stained area. Ki-67 was determined by calculating the percentage of tumor cells with positive nuclear staining based on counting 100 cells in the most intensely stained area. The expression intensity of HER2-neu was scored according to the HER-neu staining guidelines [31]. A score of 3+ was assigned for complete membranous staining in > 10% of cells, indicating a positive result. Each microarray tumor area was evaluated individually in its entirety.

##### Molecular classification

Five molecular subtypes of breast cancer were defined according to the 2011 St Gallen consensus [32].

##### Immunostaining for the EMT molecules

N-Cad, E-Cad, CD44, and Β-Cat IHC staining were scored based on the intensity and percentage of cell staining. The percentage of positive tumor cells was scored as follows: 0 = 0%; 1 ≤ 10%; 2 = 10%–50%; and 3 =  > 50%. The intensity of staining tumor cells was graded from 0 to 3 (0 = no; 1 = weak; 2 = moderate; and 3 = strong staining). The two scores were combined as follows: 0 was considered negative, 1–3 as weakly positive, 4–6 as moderately positive, and 7–9 as highly positive staining. All evaluations were made by the pathologists who were blinded to clinical information. E-cad staining was grouped as low if ≤ 6 and as high if > 7, while CD44, β-Cat, and N-Cad staining were grouped as low if ≤ 3 and as high if > 4 to ensure a balanced distribution of cases in prognostic statistical studies.

### Statistical analysis

The relationship between all prognostic parameters and biomarkers was statistically analyzed using the SPSS 29.0 program (Statistical Packages for the Social Sciences, Chicago, IL). Pearson’s Chi-Square and Fisher’s exact tests were employed to compare categorical variables. For the comparison of tumor size, number of metastatic lymph nodes, and lymph node size between pure and mixed IMK groups, the Mann–Whitney *U* test were utilized. Kaplan–Meier analysis was performed for univariate survival analyses to compare survival rates among different groups. The Cox proportional hazards model was employed to determine the variables associated with OS in the pure and mixed IMK groups.

## Results

### Cohort group characteristics

**Fig. 1:**
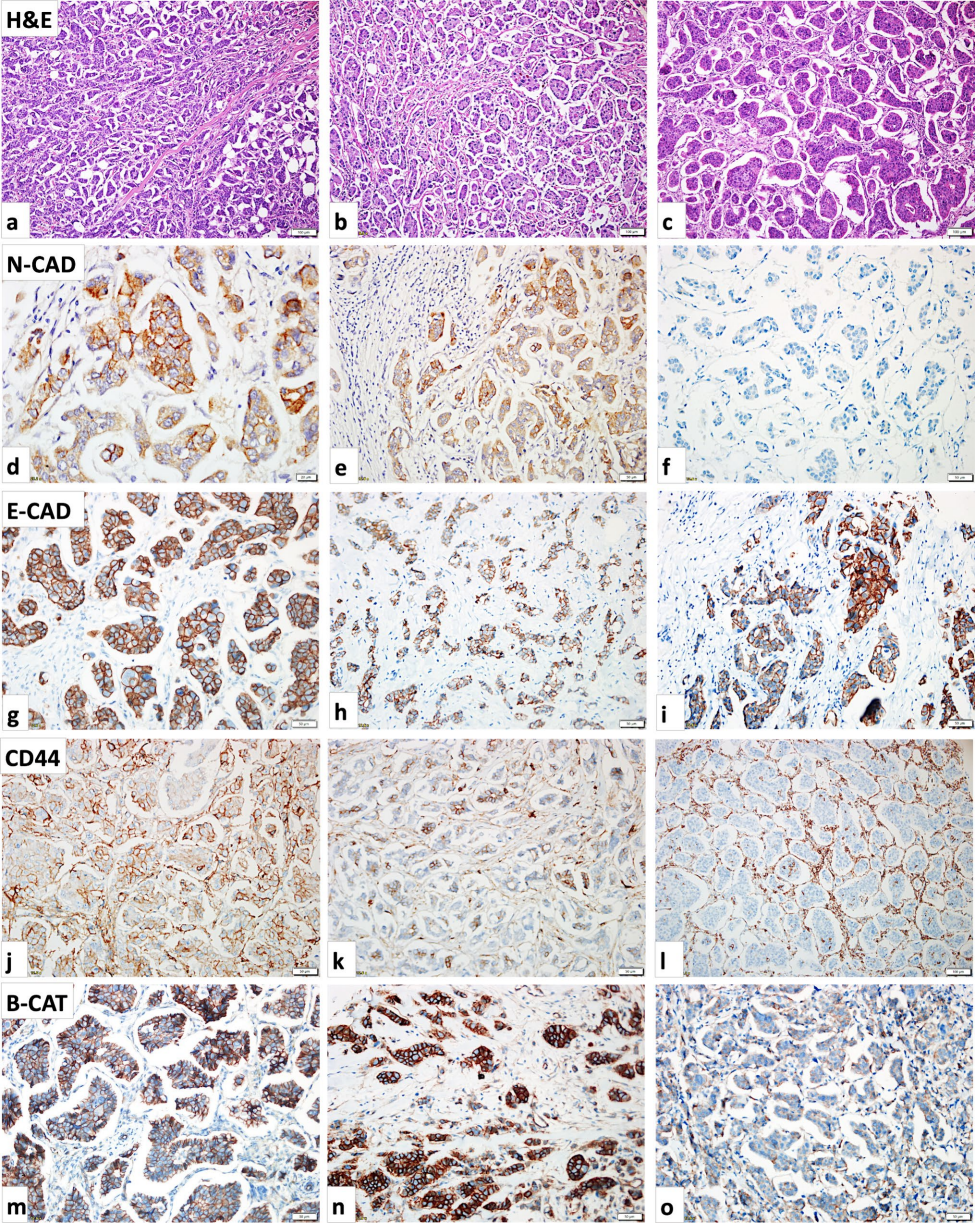
**a, b, c** H&E staining of IMPCs: showing morula-like clusters or pseudopapillary structures without a fibrovascular core of tumor cells invading the stroma. The characteristic open areas surrounding the cell clusters are striking (×10 magnification). **d, e, f** N-cadherin immunohistochemical staining of IMPCs: positive staining of N-cadherin (**d**: ×40 magnification, **e**: ×20 magnification) and negative staining of N-cadherin (**f**: ×20 magnification). **g, h, i** E-cadherin immunohistochemical staining of IMPCs: E-cadherin expression loss was not observed in any case (×20 magnification). **j, k, l** CD44 immunohistochemical staining of IMPCs. No loss of CD44 expression (**j**: ×20 magnification), partial loss of CD44 expression (**k**: ×20 magnification), and total loss of CD44 expression (**l**: ×20 magnification). **m, n, o** Beta-catenin immunohistochemical staining of IMPCs. Normal staining of Beta-catenin (**m**: ×20 magnification), cytoplasmic staining of Beta-catenin (**n**: ×20 magnification), and low membranous and weak cytoplasmic staining of Beta-catenin (**o**: ×20 magnification)

In 25 cases (25%), there were histological components of micropapillary carcinoma constituting 90% or more, leading to their classification as pure micropapillary carcinoma. The remaining 75 cases (75%) displayed micropapillary areas at varying proportions, which were less than 90% but more than 10%, classifying them as mixed IMPC. Among the mixed group, 73.3% were invasive ductal carcinoma (IDC), 12.0% were invasive lobular carcinoma (ILC), and 14.6% were a combination of IDC and ILC. Areas of ILC were diagnosed by H&E appearance and loss of e-cad expression. These micropapillary areas exhibited a brush border, indicating polarity reversal in malignant epithelial cell clusters (Fig. 1a–c). This distinctive pattern was further confirmed through EMA immunostaining.

In our study, we exclusively enrolled participants of the female gender. The median age of patients was 58.50 years (range: 31–91, mean: 58.42 ± 12.53). Twenty-nine deaths were recorded out of the cases, accounting for a mortality rate of 29.0%. The median OS time was 62.00 months (range: 2–266, mean: 78.44 ± 57.41). The median overall DFS time was 56.50 months (range: 0–266, mean: 72.54 ± 58.57). The cumulative proportions of patients overall surviving at the 12th, 24th, 48th, and 60th month were 98.0%, 92.0%, 85.0%, and 84.0%, respectively, and the cumulative proportions of patient’s disease-free surviving at the 12th, 24th, 48th, and 60th month were 94.0%, 88.0%, 54.0%, and 47.0%, respectively.

The mean tumor size was 28.54 ± 17.43 mm, with a median size of 25.00 mm (range, 7–100 cm). Furthermore, a significant association was observed between tumor size and OS time (*P* = 0.004), whereas age did not show any significant association (*P* = 0.312). Among the tumor masses, 55% were localized in the upper outer quadrant, while 88% of the tumors were localized in a single quadrant, with the remaining cases being multifocal.

Firstly, using a chi-square test, all group prognostic parameters were investigated for their association with mortality. The following factors were found to be significantly associated with mortality: the number of lymph node metastases (*P* = 0.000), pericapsular invasion (*P* = 0.028), distant metastasis (*P* = 0.000), skin involvement (*P* = 0.010), vascular embolus (*P* = 0.007), lymphatic embolus (*P* = 0.000), DFS time (*P* = 0.001), and estrogen receptor (ER) positivity (*P* = 0.001). Among the EMT markers, only N-cad (*P* = 0.007) showed a significant relationship with mortality. Furthermore, patients with clinical stages IIA and IIB (*P* = 0.000), pathological stages I and II (*P* = 0.024), and tumor-related lymphocytic infiltration (*P* = 0.020) were also found to be associated with mortality.

**Fig. 2:**
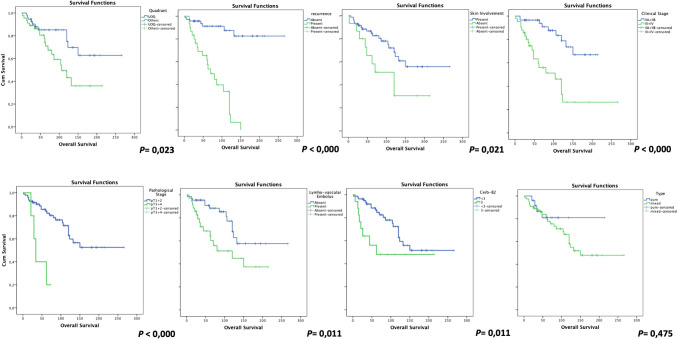
Kaplan–Meier plots of the parameters affecting prognosis in the entire IMPCs cohort group: localizations other than the upper outer quadrant (UOQ), the presence of recurrence, the skin involvement, the clinical stages 3 and 4, the pathological stages 3 and 4, the patients with lympho-vascular embolism, and the Cerb-2 score of 3 were associated with a shorter life expectancy, respectively. The expected survival time has no significant differences between pure and mixed IMPC cases (the last chart)

Survival analyses revealed that localizations other than upper outer quadrant (UOQ) (*P* = 0.023), the presence of recurrence (< 0.000), skin involvement (*P* = 0.021), clinical stages 3 and 4 (< 0.000), pathological stages 3 and 4 (< 0.000), patients with lympho-vascular embolism (*P* = 0.011), and a Cerb-2 score of 3 (*P* = 0.011) were associated with a shorter life expectancy. Notably, no significant difference in expected survival time was observed between pure and mixed IMPC cases (*P* = 0.475). Kaplan–Meier graphs are demonstrated in Fig. 2. Moreover, no significant impact of other parameters on the expected OS was detected.

All patients underwent breast-conserving surgery or modified radical mastectomy. Axillary lymph node dissection was performed on patients with four positive nodal metastases. It was observed that patients who underwent modified radical mastectomy and sentinel lymph node dissection had a significantly longer survival time (*P* = 0.002). None of the patients received neoadjuvant chemotherapy. There was no significant difference in adjuvant chemotherapy between the two groups, and all patients received adjuvant radiation therapy (RT) as per the guidelines.

### The comparison of pathological characteristics of pure and mixed IMPCs

Tumor size was 35.5 ± 24.2 mm in pure cases and 26.2 ± 13.8 mm in the mixed cases, with no significant difference detected (*P* = 0.137). The mean number of metastatic lymph nodes was 6.9 ± 10.6 in pure cases and 5.1 ± 6.0 in mixed cases, and no significant difference was found (*P* = 0.945). According to the general histological evaluation of the tumor tissue, most patients were classified as histological grade 3, with 92.0% in the pure IMPC cases and 67.6% in the mixed IMPC cases (*P* = 0.016). Furthermore, significant differences were observed in the ratio of the Intraductal component (*P* < 0.001), recurrence frequency (*P* = 0.023), pathological stage (*P* = 0.013), and tumor-related lymphocyte intensity (*P* = 0.002). However, no significant differences were observed in the other prognostic parameters, including molecular and EMT marker staining patterns (*p* > 0.05). A comparison of the pathological characteristics of pure and mixed IMPCs is presented in Table 1.

### The comparison of differences in immunohistochemical molecular classification of Pure and mixed IMPCs

Among the pure IMPC cases, 3 cases (12.0%) were classified as Luminal A, 14 cases (56.0%) as Luminal B, 2 cases (8.0%) as Luminal B and HER2, 5 cases (20.0%) as HER2 rich, and 1 case (4.0%) as triple negative. In the 75 mixed IMPC cases, 15 cases (20.0%) were classified as Luminal A, 32 cases (42.6%) as Luminal B, 20 cases (26.6%) as Luminal B and HER2, 7 cases (9.4%) as HER2 rich, and 1 case (1.4%) as triple negative. No statistically significant differences were found between the Luminal A and B subgroups, Luminal A and HER2-rich subgroups, and Luminal B and HER2-rich subgroups, with respective *p*-values of 0.353, 0.210, and 0.502. Further analysis of molecular classification members using Pearson Chi-square and Cox survival analysis also did not reveal any differences among the subgroups (*P* > 0.05) (Tables 1 and 2). In terms of OS, it was observed that only a Cerb-2 score < 3 (*P* < 0.001) was significantly associated with OS and posed no risk for short survival in the mixed IMPC group. However, this association was not observed in the pure IMPC group (*P* = 0.991) (Table 2).

**Table 2:** The effect of prognostic parameters and EMT biomarker expression intensity with OS time in pure and mixed invasive micropapillary carcinoma (IMPC) cases

Parameters	Mixed				Pure			
	HR	95% CI		*P*	HR	95% CI		*P*
		Lower	Upper			Lower	Upper	
Quadrant (ref: upper outer)	2.458	1.093	5.526	**0.030**	2.858	0.296	27.552	0.364
Pericapsular invasion (ref: absent)	1.251	0.562	2.789	0.583	NA	NA	NA	NA
Distant recurrence (ref: absent)	10.193	4.159	24.986	** < 0.001**	NA	NA	NA	NA
Skin involvement (ref: absent)	2.239	0.888	5.645	0.087	9.642	1.002	92.759	**0.050**
Pathological category (ref: pT1&2)	4.740	0.614	36.615	0.136	24.469	2.448	244.563	**0.006**
Clinical stage (Ref: IIA and IIB)	3.804	1.671	8.661	** < 0.001**	NA	NA	NA	NA
Tumor-infiltrating lymphocytes (ref: low)	2.013	0.235	17.231	0.523	NA	NA	NA	NA
Lympho-vascular embolus (ref: absent)	2.213	1.008	4.858	**0.048**	7.766	0.786	76.732	0.079
Tumor size (ref: ≤ 20 mm)	5.497	1.962	15.406	** < 0.001**	1.770	0.183	17.165	0.622
Number of the LNM (ref: < 4)	3.916	1.699	9.025	** < 0.001**	7.766	0.786	76.732	0.079
Lymph node size (ref: < 17 mm)	2.768	1.134	6.753	**0.025**	0.529	0.055	5.099	0.582
Ratio of intraductal component (ref: < 5%)	0.551	0.204	1.487	0.239	12.223	1.238	120.715	**0.032**
ER (ref: negative)	0.422	0.155	1.146	0.091	0.824	0.085	7.957	0.867
PR (ref: negative)	0.447	0.182	1.097	0.079	1.550	0.161	14.924	0.705
Ki67 (ref: < 14%)	0.727	0.188	2.818	0.645	NA	NA	NA	NA
Cerb-2 (ref: score < 3)	4.924	2.012	12.055	** < 0.001**	1.013	0.105	9.766	0.991
B-CAT (ref: low)	0.857	0.320	2.293	0.759	0.427	0.044	4.114	0.462
CD44 (ref: low)	0.677	0.305	1.503	0.338	0.023	0.000	109.815	0.383
N-CAD (ref: low)	1.592	0.724	3.502	0.248	14.990	1.543	145.659	**0.020**
E-CAD (ref: low)	1.981	0.743	5.281	0.172	0.884	0.092	8.516	0.915

*The P values < 0.05 are bolded

### The comparison of survival outcomes of pure and mixed IMPCs

**Fig. 3:**
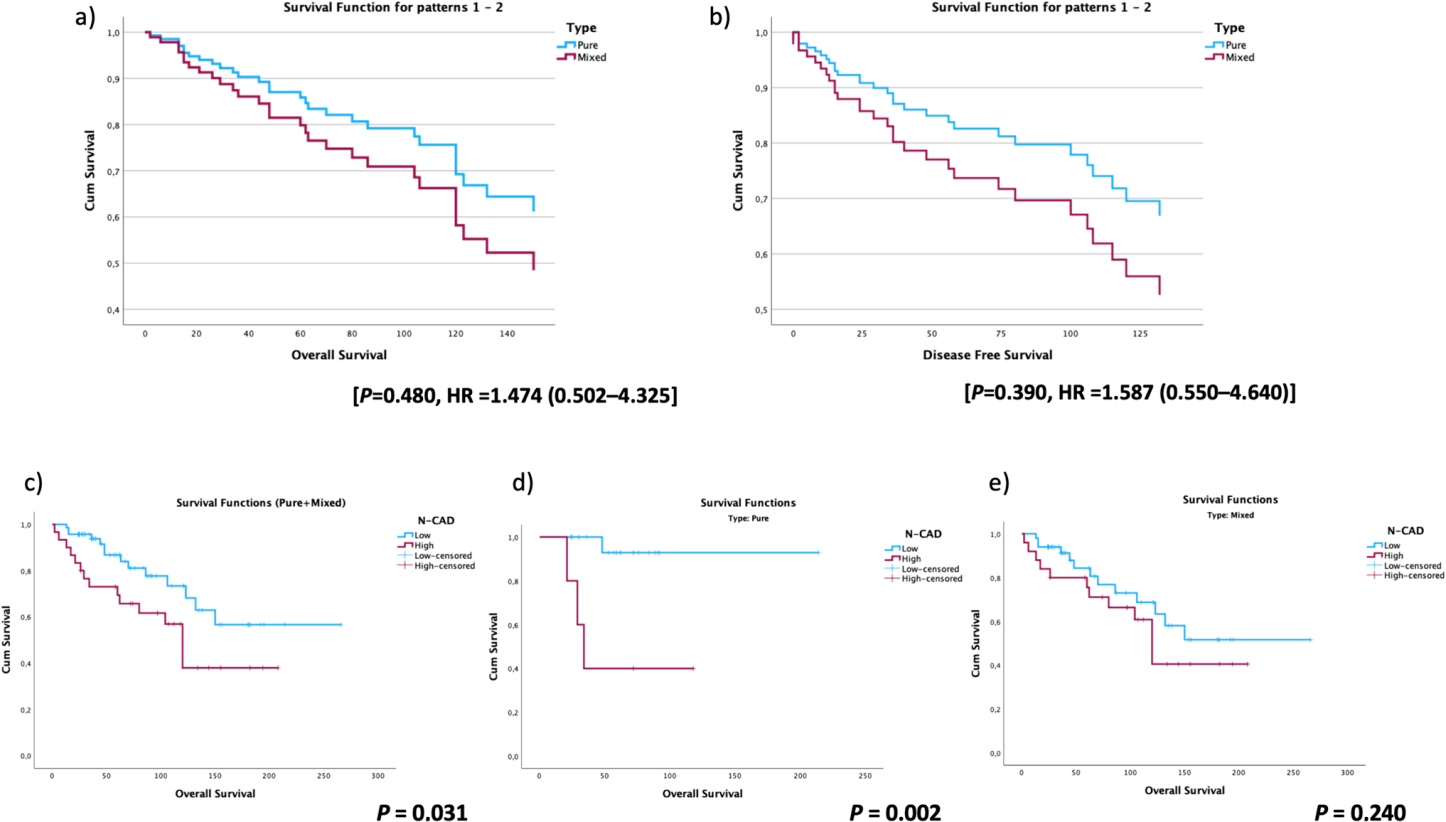
The comparison analyses of OS, DFS between pure and mixed IMPC groups, and N-cadherin survival plots in entire, pure, and mixed groups: **a, b** The OS and DFS cox proportional hazards plots between pure and mixed IMPCs groups. **c, d, e** Kaplan–Meier plots of low and high N-cadherin expressions in the entire (**c**), pure (**d**), and mixed (**e**) IMPCs groups

The mortality rates were 16.0% (4/25) in the pure IMPC group and 33.3% (21/75) in the mixed IMPC group. The estimated total survival time was 179.72 ± 15.54 months in the pure IMPC group and 168.85 ± 14.39 months in the mixed IMPC group. No significant difference was found between the two groups (*P* = 0.475) (Fig. 2). In the comparative survival analysis of the whole group based on OS and DFS for pure and mixed groups showed no significant difference, with a *P* value of 0.480 and 0.390, respectively, and hazard ratios (HR) of 1.474 (0.502–4.325) and 1.587 (0.550–4.640), respectively (Fig. 3a, b).

The 2-year DFS rates were 72.0% and 76.0% in the pure and mixed IMPC groups, respectively, and the 3-year DFS rates were 56.0% and 61.3%, respectively (*P* = 0.208) (Table 1).

Although, the upper outer quadrant localization (*P* = 0.030), absence of recurrence (*P* < 0.001), clinical stages IIA&IIB (*P* < 0.001), absence of LVI (*P* = 0.048), tumor size ≤ 20 mm (*P* < 0.001), number of the LNM < 4 (*P* < 0.001), and the largest size of metastatic lymph node < 17 mm (*P* = 0.025) and Cerb-2 score < 3 (*P* < 0.001) were associated with OS and did not pose a risk for short survival in the mixed IMPC group, none of these parameters were associated with OS in the pure IMPC group (Table 2).

Only absent of LVI and the number of the LNM < 4 showed a relatively weak association with OS in the pure IMPC group (*P* = 0.079, 0.079, respectively). Conversely, skin involvement (*P* = 0.050), pT3&4 category (*P* = 0.006), the ratio of intraductal component (> 5%) (*P* = 0.032), and N-cad high-level expression (*P* = 0.020) in pure IMPC patients were associated with a higher risk of short survival in the pure IMPC group (Table 2).

### The comparison of pure and mixed IMPC cases with expression intensity of the EMT markers accompanied by survival analysis (OS-DFS)

The thirty cases with high N-cad expression (30.0%) in our cohort group demonstrated a shorter OS time (*P* = 0.031). N-cad IHC immunostaining positive and negative photo results are demonstrated in Fig. 1d–f. Five of the thirty cases in the pure IMPC group exhibited a shorter OS time (*P* = 0.002). However, in the mixed IMPC group, twenty-five out of thirty cases did not show a shorter OS time (*P* = 0.240) (Table 3). Figure 3 illustrates the corresponding Kaplan–Meier graphs of N-cad and OS time (Fig. 3c–e). Furthermore, in comparison, the cohort group (*P* = 0.008) and pure IMPC group exhibited a shorter DFS time (*P* = 0.001); however, the mixed IMPC group did not show a shorter DFS time (*P* = 0.122) (Table 3). Cox-regression survival analyses were conducted to evaluate the N-cad expression density. The results revealed that cases with N-cad overexpression in the pure IMPC subgroup had approximately 15 times higher mortality risk (*P* = 0.020). Conversely, no significant mortality risk was observed in the mixed IMPC cases (Table 2) (*P* = 0.248). There was no detected E-cad expression losing in any tumor samples, and E-cad IHC immunostaining photo results are demonstrated in Fig. 1g–i. The CD44 IHC immunostaining positive and negative photo results are demonstrated in Fig. 1j–l. The normal β-cat IHC staining result (Fig. 1m) and differences have been detected as gaining cytoplasmic staining (Fig. 1n) and losing membranous staining (o) are demonstrated. There was no discernible association between the expression intensities of β-cat, CD44, and E-cad markers in pure, mixed, and all cohort IMPC cases and OS/DFS or any risk differences among the groups (*P* > 0.05) (Table 3).

**Table 3:** The results of EMT biomarkers in pure, mixed, and all cohort invasive micropapillary carcinoma (IMPC) cases and their relationship with OS and DFS time (Kaplan–Meier/Mouth)

Variables	Pure and mixed IMPC		Pure IMPC		Mixed IMPC	
	Overall survival	*P* value^a^	Overall survival	*P* value^a^	Overall survival	*P* value^a^
*B-CAT*						
Low	107.72 ± 15.19	0.560	88.3 ± 24.2	0.448	109.06 ± 16.38	0.757
High	175.40 ± 14.59		183.9 ± 16.0		170.73 ± 15.75	
*CD44*						
Low	144.73 ± 19.91	0.162	^b^	0.124	144.24 ± 21.22	0.332
High	160.13 ± 12.84				140.84 ± 12.53	
*N-CAD*	189.25 ± 16.86	**0.031**	202.1 ± 11.4	**0.002**	178.48 ± 18.68	0.240
Low	119.34 ± 15.53		64.0 ± 19.8		126.22 ± 16.50	
High						
*E-CAD*						0.161
Low	194.3 ± 26.04		63.4 ± 7.7		195.69 ± 27.04	
High	140.04 ± 11.34		179.9 ± 17.9		132.00 ± 12.07	

	Disease-free survival		Disease-free survival		Disease-free survival	
*B-CAT*		0.501		0.368		0.691
Low	107.55 ± 15.84		78.67 ± 32.12		107.84 ± 17.03	
High	176.75 ± 15.00		184.34 ± 15.87		169.81 ± 16.39	
*CD44*		0.251		0.139		0.476
Low	146.78 ± 21.23				142.52 ± 22.58	
High	158.06 ± 13.50				136.47 ± 12.48	
*N-CAD*		**0.008**		**0.001**		0.122
Low	192.85 ± 16.68		203.58 ± 10.14		181.07 ± 18.67	
High	112.04 ± 17.13		44.10 ± 24.81		118.26 ± 18.27	
*E-CAD*		0.258		0.805		0.150
Low	200.99 ± 24.84		57.6 ± 12.88		201.275 ± 25.72	
High	138.63 ± 12.01		180.99 ± 17.51		127.77 ± 12.95	

^a^Long-rank

^b^Low mortality rate

*The P values < 0.05 are bolded

## Discussion

It is crucial to acknowledge that prognosis in IMPC is influenced by various factors, such as tumor stage, grade, hormone receptor status, HER2/neu expression, and patient characteristics. IMPC remains classified under the subgroup with a poor prognosis in the WHO 2019 classification [4]. The micro-papillary component ratio is among the several factors taken into account when evaluating prognosis and should be interpreted in conjunction with other clinicopathological variables.

The prevalence of the micro-papillary component within a tumor mass can offer valuable insights into its behavior. Several publications have indicated that a higher percentage of micro-papillary components within the tumor is associated with an increased risk of lymph node metastasis [6, 11–13, 18], distant metastasis, and poorer OS [3, 11, 12]. However, it has also been argued that the extent of the micropapillary component does not significantly affect prognosis, as any proportion of IMPC still exhibits a higher incidence of LVI and LNM [8, 10, 14, 19].

Furthermore, recent studies have suggested that the prognosis of IMPC may not be worse than that of invasive breast carcinoma of no special type (IBC NST), despite its clinically unfavorable characteristics [5, 9, 13, 33]. This discrepancy in the existing literature might be attributed to the lack of a clear definition for pure IMPC cases, emphasizing the need for precise quantification of the micro-papillary involvement within the tumor mass to obtain valuable prognostic information. Currently, there is no universally agreed-upon threshold or cut-off value for the percentage or ratio of micro-papillary components that determines the prognosis of breast carcinoma. Some studies consider cases as “Pure IMPC” when the micro-papillary component constitutes over 75% of the tumor mass [8, 11, 12, 18, 19, 33, 34], while others accept it at over two-thirds (66%) [10] or even above 50% [13, 14]. Rarely, a grouping of 90% or higher has been achieved [17, 18]. A study considering a rate above 90% has reported a shorter DFS for pure IMPC than for the mixed group [18].

The issue of defining breast cancer subtypes has been effectively addressed in the 5th WHO classification. In this classification, mixed IBC-NST is described when the special type carcinoma component is present at a rate of 10–90%. However, the classification does not consider cases where the specific types account for less than 10%. Pure breast carcinoma, on the other hand, is diagnosed when the predominant component constitutes more than 90% [3, 4].

In our study, we propose the classification of cases based on the concept that pure IMPC can be considered as a distinct entity. Following the guideline the World Health Organization set fifth in 2019, we categorized our cases using a > 90% IMPC component threshold. The criterion of > 90% ratio for pure IMPC has been mentioned in a few studies, such as the ones conducted by Zekioğlu et al. and Küpik et al. [11, 18], where it was defined as the presence of a complete micropapillary component. However, the existing literature has not clearly established this ratio definition.

The incidence rate of IMPC cases within our cohort group was found to be 4.68%. This rate aligns with the range of 2% to 8% reported in previous literature, as documented [8, 11, 17, 35]. Furthermore, studies examining mixed invasive breast cancers containing IMPC components have reported the presence of micropapillary growth patterns in focal areas, with rates ranging from 3 to 8.4% [7, 8, 10, 14, 17]. Upon considering cases that were classified as pure IMPC in our work, we observed a prevalence rate of 0.6%, which is lower than the reported pure rates in the literature. It has been reported that if the IMPC component ratio exceeds 75%, the prevalence of IMPC among all cases of IBC-NST decreases to a range of 0.7% to 2%. [4, 5, 8, 11, 14, 17, 35].

Given the uncertainty surrounding the literature findings on the rate of micropapillary components and prognostic parameters, it is imperative to classify our discoveries and engage in a comprehensive discussion based on the existing body of literature.

### Lymph node involvement, distant metastasis, OS, and DFS

In the comparative survival analysis conducted on the entire group, no significant difference was observed between the pure and mixed groups (Fig. 3a, b; Table 2). When comparing our findings, it would be useful to remember that it is necessary to keep in mind the differences in the ratio of micropapillary components in different studies. Our findings concerning OS and DFS align with the studies conducted by Ide et al. and Kaya et al. [10, 19]

In our cohort group, we observed an LVI rate of 57.3% and a rate of 77.2% for LNM when considering all cases of IMPC. These findings align with the rates reported in the literature, which range from 78.7 to 94.7% for LVI and 72.3 to 79.6% for LN metastasis [14, 19].

Specifically, when we analyzed pure cases of IMPC, the LVI rate was 62.7%, while it was 52.0% for mixed cases. These rates are consistent with previous studies that compared IMPC with IBC-NST [6, 7, 10, 11, 14, 15, 19]. Furthermore, when we compared our results with studies focusing on IDC cases, we found that the LVI rates in IMPC were higher [6, 7]. Importantly, we did not observe a significant difference in LVI rates between pure and mixed IMPC groups (Table 1) [14, 19].

In our group of locally advanced IMPC cases, an examination of those with more than four positive lymph nodes (LNM > 4) revealed a significant 3.9-fold increase in mortality rate (< 0.001). Additionally, cases with lymph node size exceeding 17 mm showed a 2.7-fold increase in mortality rate (*P* = 0.025). However, when considering these parameters, no significant effect on survival was observed in the pure IMPC group (*P* = 0.079, 0.582, respectively). These findings are consistent with the results reported by Lewis et al. [9] but contradict the findings of Kupik et al. [18]. The lack of prognostic impact of the number and size of lymph node metastases in the pure IMPC group suggests that this subgroup behaves differently from the mixed IMPC group, although no discernible prognostic difference between the two groups was detected.

The pure group exhibited a distant recurrence rate that was 2 times higher compared to the mixed group (*P* = 0.023). This finding contradicts previous studies conducted by Gokce et al., where a pure identification rate of 75% or higher was reported [14] and Kupik et al. [18]. Additionally, cases with recurrence in the mixed group had a mortality risk that was 10.1 times higher (< 0.001), while no conclusive result could be obtained for the pure group due to its low mortality rate. These results support the conclusion that the IMPC component increases the risk of morbidity, even in areas with IMPC component proportions below 90% [8, 10, 19].

It has been reported that there was no significant difference in 3-year DFS between cases of pure IMPC and cases with mixed IMPC. However, pure IMPK cases were associated with shorter OS [13]. Kupik et al. also observed shorter DFS rates in pure cases of IMPC within their series, where the entire micropapillary component was considered pure, compared to the mixed group; however, they did not provide a specific ratio. They did not find a difference in OS between the two groups [18]. Our findings regarding OS are consistent with Kupik et al., Gokce et al., and Wang et al. [13, 14, 18]. However, our findings regarding DFS contradict those of Wang et al., Kupik et al., and Gokce et al. [13, 14, 18].

### Hormone receptor status and molecular subtypes

IMPCs generally exhibit ER+ and PR+ characteristics [4, 11, 14, 17]. Consequently, they are treated following the standard treatment protocol for IBC-NST. Previous studies have reported that hormone-positive IMPC cases have a favorable prognosis, similar to that of IBC-NST cases [6, 7, 14].

In our cohort case group, the molecular subclassification revealed that 18.0% of the cases were classified as Luminal A, 46.0% as Luminal B, and 22.0% as Luminal B and HER2. Out of 100 cases, 86 (86.0%) were found to be ER+, consistent with the literature [6, 7, 15, 36, 37].

Statistical analysis did not reveal significant differences between the Luminal A and B subgroups, Luminal A and HER2-rich subgroups, and Luminal B and HER2-rich subgroups in both pure and mixed IMPC cases. Contrary to the findings of Wang et al., who reported a higher proportion of Luminal B in the pure group compared to the mixed IMPC group, our results were inconsistent with theirs [13].

On the contrary, although only estrogen receptor (ER) positivity was found to be associated with mortality in the entire cohort group [4, 11], this significance could not be demonstrated in the comparative survival analyses conducted between pure and mixed groups [10, 13, 18, 19]. Moreover, the ER receptor status did not show any association with survival in the Kaplan–Meier analyses of the entire cohort group (*P* = 0.562). There was no discernible difference observed in the comparative analyses of ER, PR, and Ki-67 biomarkers in pure and mixed groups (Table 1), as well as in the comparative survival analyses based on ER, PR negative, and Ki-67 < 14% (Table 2). These findings are inconsistent with the literature data suggesting that ER receptor positivity serves as a favorable prognostic indicator in IMPC [6], and they support the notion that IMPC represents a hormone-independent subgroup of breast cancers [11].

### HER2/neu status

There are conflicting reports regarding the rate of HER2-positive cases, as discussed in some studies [4, 11, 36]. It has been accepted that HER2 positivity is indicated only when scoring 3 for Cerb-2 IHC staining. In our study, out of the entire group, 34 cases (34.0%) were determined as HER2 positive, regardless of hormone status. Similar rates have been reported in the literature, such as the study by Tang et al. [15, 17]. However, our HER2-positive ratio is slightly higher compared to some larger series [36, 37]. The HER2 positive ratio is higher than the IBC-NST and consistent with some studies [15, 36].

Among the mixed group of patients, comprising 27 individuals (36.0%) who scored HER2 positive, we found that their life expectancy was 4.9 times shorter (< 0.001). However, this result was not observed in the pure group of 7 patients (28%) (*P* = 0.991).

Our incidence rate of IMPC with triple-negative (TN) subtype was only 2%, which prevented us from obtaining statistically significant results due to the limited number of cases. The prevalence of the TN subtype in IMPC is estimated to be approximately 5%, and it is widely acknowledged as having the poorest prognosis among the reported groups [36]. Consequently, it is deemed appropriate to classify and treat these cases as TN IBC-NST.

### Pathological stage and histological grade

Higher-grade IMPCs may be associated with a poorer prognosis. Pathological stage 3&4 was found to have a 12.30 times higher mortality risk in the pure group. Furthermore, histological grade 3 cases were more prevalent in the pure group, indicating a significant difference between the two groups (*P* = 0.016). Notably, in the histological grade 3 cases, the mortality risk was found to be 24.5 times higher in the pure group. These findings deviate from the existing literature [14, 19].

### Ki-67 proliferation index

A higher Ki-67 proliferation index is typically associated with a poorer prognosis in IMPC. The pure group displayed a presence of 64.0% (including Luminal B and HER2+ at 8.0%), while the mixed group showed a presence of 69.2% (including Luminal B and HER2+ at 26.6%). These findings indicate a generally high proliferation index in IMPC, with no significant differences observed between the pure and mixed IMPC groups. Notably, these results contradict the findings of Wang et al., who reported a higher proportion of Luminal B in pure IMPC compared to mixed IMPC [13]. In addition, when compared to IBC-NST, both pure and mixed IMPC groups exhibited similar rates of proliferation index, which aligns with the existing literature [17].

### Tumor-related lymphocyte infiltration

The rate of tumor-related lymphocytes was generally high in the entire group, but it was detected in all cases of the pure group, indicating a significant difference between the two groups (*P* = 0.002) (Table 1). The mixed group had no observed relationship between TIL density and survival analysis (*P* = 0.523). However, the pure group could not achieve statistical significance due to insufficient cases resulting in a low mortality rate. Interestingly, these findings align with the results reported by Deman et al. [34].

### Intraductal component ratio

In the pure group, the ductal carcinoma in situ occurrence rate is extremely low, almost non-existent, which differs significantly from the mixed group (< 0.001). Additionally, when the intraductal component exceeds 5%, the mortality rate increases by a factor of 12.2 in the pure group. However, we were unable to obtain literature information regarding this data.

### Tumor size

In cases with mixed characteristics, there was a notable 5.5-fold increase in the mortality rate (< 0.001) for tumors larger than 20 mm. However, tumor size did not influence the prognosis in pure cases. The average tumor size was 56.78 mm for pure IMPC cases and 47.01 mm for mixed cases. Despite the relatively larger tumor size observed in pure cases, no statistically significant difference was observed in the comparative and survival analyses (*P* = 0.356, *P* = 0.137, respectively) [13, 18, 19].

### Others

In pure cases, skin involvement was found to increase the mortality risk by 9.6 times. In mixed cases, the mortality rate showed an increase of 3.8 times in patients with clinical stages 3&4 and 2.2 times in cases with (Table 2).

Comparative statistical analyses did not reveal any significant differences in terms of mortality ratio, age, tumor size, number of metastatic lymph nodes, largest lymph node size, number of tumoral masses, lymph node pericapsular invasion, distant metastasis, skin involvement, clinical stage, modified Scarff–Bloom–Richardson histological grading (tubular formation, nuclear grade, mitosis), surgical margin, vascular embolus, nerve invasion, OS, or DFS (Table 1).

### The differences in the molecular level regarding metastasis mechanisms in pure and mixed invasive micropapillary breast carcinoma

Our analysis of the N-cad biomarker revealed that increased expression of N-cad in the pure IMPC group was associated with significantly shorter OS and DMFS. Similar results were obtained when analyzing the entire cohort group for OS and DMFS with N-cad. However, when we specifically looked at the group of mixed IMPC cases, we did not observe the same significant associations for OS and DMFS (Table 3). N-cad and OS Kaplan–Meier graphs are demonstrated in Fig. 3c–e.

Furthermore, while increased N-cad expression was associated with a 14.9-fold higher mortality risk in the pure group, such an association was not found in the mixed group (Table 2). These findings suggest that the prognostic effect of N-cad observed in the entire cohort group is primarily driven by its impact on pure IMPC cases. This molecular-level evidence supports the notion that pure and mixed IMPC are distinct entities with different clinical behaviors. Although there is limited literature on the role of N-cad as a marker distinguishing between pure and mixed IMPC, our findings are consistent with previous studies, such as Nagi et al., which demonstrated the prognostic importance of N-cad in distinguishing between IMPC and IBC-NST[26].

In our series, we found higher expression of N-cad, a mesenchymal cell marker associated with EMT, in pure IMPC cases, aligning with existing literature suggesting that the IMPC component has a higher metastatic potential [3, 5, 12, 13]. However, despite the increased N-cad expression and higher metastatic potential observed in pure IMPC, the expected life expectancy is not shorter compared to mixed IMPC.

In the survival analyses of E-cad, β-cat, and CD44 markers, as well as in both pure and mixed patient groups, no significant effect on survival was observed (Table 3). Furthermore, in comparative analyses, no significant differences were found in the expression levels of these biomarkers between the two groups (Table 1). These findings suggest that the lack of change in E-cad expression in tightly attached groups may be expected, indicating that IMPC cancer cells maintained their epithelial properties. Notably, the increase in N-cad expression observed in the Pure IMPC group aligns with previous findings in basal-like or high-grade breast cancers [22, 27].

The obtained results provide potential evidence indicating that the molecular mechanisms involved in the process of EMT may exhibit distinct arrangements in pure IMPC compared to mixed IMPC. No other studies conducted in a similar manner have been found in the existing literature. Nevertheless, these findings exhibit similarities to previous studies exploring EMT-related molecules and genetic biomarkers [16].

Despite numerous studies in the literature investigating prognostic differences between pure&mixed and aggressive clinical features of IMPC [10, 12–14, 18, 19], it remains unclear whether the presence of IMPC leads to reduced OS compared to IBC-NST [6, 7, 9, 11, 15, 17]. Recent evidence suggests a diminishing gap in prognosis between IMPC and IBC-NST, with reports indicating no differences in cell polarity and adhesion-related gene expressions [37].

To summarize the findings, skin involvement, low intraductal component, high pathological stage, and increased N-cad expression in the pure IMPC group were found to have a more detrimental impact on survival rates compared to the mixed group in survival analyses. Due to a low mortality rate in the pure group, no conclusive results could be obtained regarding the presence of pericapsular invasion, distant recurrence, and high clinical stage. Interestingly, the pure group showed no significant association with LVI, tumor size, number of involved lymph nodes, metastatic lymph node size, or HER2 positivity, which affected survival in the mixed group. Despite these differences, no distinct prognostic disparities were observed between the two groups.

The number of cases in our study is limited due to the accepted ratio for pure IMPC. In line with our study objectives, our findings support the notion that pure IMPC can be considered a distinct entity, characterized by molecular level differences and a tendency for more aggressive clinical behavior compared to the mixed group. Although we did not observe any differences between the two groups in terms of OS and DFS, this does not invalidate the hypothesis that these groups possess distinct characteristics.

It is important to emphasize that this study defines pure IMPC as 90% or more in composition, and these findings are noteworthy given the limited data available in the literature on this subject. The detection of increased N-cad expression in the locally advanced IMPC group, aiding in the differentiation between pure and mixed IMPC, lends support to the involvement of EMT mechanisms in the pure IMPC group. These findings will serve as a foundation for further studies.

We firmly believe that our study will significantly contribute to the existing literature by discussing the proportion of micropapillary components in pure and mixed IMPC cases, a rare subtype of breast carcinoma with high metastatic potential. Moreover, our research displayed these two groups’ molecular differences regarding some EMT mechanisms. Our study is qualified to lead the studies to be planned in extensive series.

## Supplementary Information

Below is the link to the electronic supplementary material.

Supplementary file1 (DOCX 21 KB)

## Data Availability

Inquiries about data availability should be directed to the corresponding author.
