# Supplementary material for: The differences between pure and mixed invasive micropapillary breast cancer: the epithelial–mesenchymal transition molecules and prognosis
Source: Breast Cancer Res Treat. 2024 Jul 2;208(1):41–55. doi: 10.1007/s10549-024-07384-w (PMC11452530; doi:10.1007/s10549-024-07384-w)
Supplement: Supplementary file 1 — Supplementary file1 (DOCX 21 KB) [file 10549_2024_7384_MOESM1_ESM.docx]

| TABLE . Biomarkers used in the study by Immunohistochemical (IHC) method | | | |
| --- | --- | --- | --- |
| **Antibodies** | ***Source*** | ***Clone*** | ***Dilution*** |
| **CD44s** | **Dako** | **DF1485** | **1/50** |
| **E-cad** | **Dako** | **NCH-38** | **Ready to use** |
| **N-cad** | **Santa Cruz** | **H-2** | **1/100** |
| **β-cat** | **Dako** | **β-Catenin-1** | **Ready to use** |
| **ER** | **Dako** | **EP1** | **Ready to use** |
| **PR** | **Dako** | **PgR 636,** **PgR 1294** | **Ready to use** |
| **HER-2** | **Dako** | **Polyclonal (A0485)** | **1:1200** |
| **Kİ-67** | **Dako** | **MIB-1** | **Ready to use** |
